# Trypanosomiasis-Induced Megacolon Illustrates How Myenteric Neurons Modulate the Risk for Colon Cancer in Rats and Humans

**DOI:** 10.1371/journal.pntd.0003744

**Published:** 2015-04-17

**Authors:** Vinicius Kannen, Enio C. de Oliveira, Bruno Zene Motta, Annuar Jose Chaguri, Mariângela Ottoboni Brunaldi, Sérgio B. Garcia

**Affiliations:** 1 Department of Pathology, University of Sao Paulo, Ribeirao Preto, Brazil; 2 Department of Surgery, Medical School, Federal University of Goias, Goiania, Brazil; Federal University of São Paulo, BRAZIL

## Abstract

**Background:**

Trypanosomiasis induces a remarkable myenteric neuronal degeneration leading to megacolon. Very little is known about the risk for colon cancer in chagasic megacolon patients. To clarify whether chagasic megacolon impacts on colon carcinogenesis, we investigated the risk for colon cancer in *Trypanosoma cruzi* (*T*. *cruzi*) infected patients and rats.

**Methods:**

Colon samples from *T*. *cruzi*-infected and uninfected patients and rats were histopathologically investigated with colon cancer biomarkers. An experimental model for chemical myenteric denervation was also performed to verify the myenteric neuronal effects on colon carcinogenesis. All experiments complied the guidelines and approval of ethical institutional review boards.

**Results:**

No colon tumors were found in chagasic megacolon samples. A significant myenteric neuronal denervation was observed. Epithelial cell proliferation and hyperplasia were found increased in chagasic megacolon. Analyzing the argyrophilic nucleolar organiser regions within the cryptal bottom revealed reduced risk for colon cancer in Chagas’ megacolon patients. *T*. *cruzi*-infected rats showed a significant myenteric neuronal denervation and decreased numbers of colon preneoplastic lesions. In chemical myenteric denervated rats preneoplastic lesions were reduced from the 2^nd^ wk onward, which ensued having the colon myenteric denervation significantly induced.

**Conclusion/Significance:**

Our data suggest that the trypanosomiasis-related myenteric neuronal degeneration protects the colon tissue from carcinogenic events. Current findings highlight potential mechanisms in tropical diseases and cancer research.

## Author Summary

The myenteric neuronal activity on colon carcinogenesis is a matter of debate. Chagas disease (a trypanosomiasis-related chronic infection) induces megacolon damaging myenteric neurons. Puzzling, tumors have been rarely reported in chagasic megacolon patients. We reveal here hyperplasia-related high-proliferation occurs in chagasic megacolon, although the risk for colon cancer is reduced. Having carcinogen-exposed rats infected with *Trypanosoma cruzi* reduced the numbers of myenteric neurons and colon preneoplastic lesions. An experimental model for chemical myenteric denervation was applied in carcinogen-exposed rats revealing that myenteric neurons promote the development of colon preneoplastic lesions. Yet, activity of the fecal content had to be secluded from the myenteric neuronal activity on colon carcinogenesis. Hartmann’s surgical procedure enabled that. This was applied together with carcinogenic exposure and myenteric neuronal denervation ensuring that the neuronal activity is associated with enhanced development of colon carcinogenesis. Taken together, we believe colon tumors are not found within the chagasic megacolon region because the myenteric neuronal density is impaired. These observations shed lights on novel potential cell to cell interactions promoting the colon cancer development.

## Introduction

Over a century ago the Brazilian physician Carlos Chagas described the American trypanosomiasis, named after him the “Chagas disease” [1]. Fritz Koberle, one of the very founders of our department, investigated for decades the Chagas disease hypothesizing the “neurogenic theory” [2]. While Chagas had previously observed that the brainstem undergo inflammatory degeneration due to the *Trypanossoma cruzi* (*T*. *cruzi*) infection [1], Koberle proposed the *T*. *cruzi*-related degeneration of the peripheral nervous system promote the “gross enlargement of the oesophagus, colon, and heart” [2]. A careful reading of the original Chagas’ manuscript reveals he might catch a glimpse of the relationship between the trypanosomiasis-induced neuronal degeneration and Chagas heart disease, which, in fact, was proven by Koberle [1,2].

Major health concern lies on the poor living conditions of those 8 to 10 million American trypanosomiasis-infected patients. Regardless of the fact that neither all infection cases show symptoms nor all acute patients develop the chronic disease, about 12,500 people die annually by Chagas disease in Latin America [3,4]. Great efforts have been launched to control new cases of the Chagas disease in the whole American continent, from which “The London Declaration on Neglected Tropical Diseases” is the most recent initiative [5].

Although *T*. *cruzi*-infection is a life-threatening condition, scientific research has shown new potential avenues for cancer therapy understanding this parasite activity on the immune system [6,7]. A 47-kDa chaperone that has been named *T*. *cruzi* calreticulin (TcCRT) binds to the C1 complement element and the mannan-binding lectin (MBL) modulating immune response and angiogenesis [7,8]. Interestingly, *T*. *cruzi*-related modulatory activity on immune system has been reported to decrease not only tumor growth, but also cancer-related angiogenesis [8,9].

For the last fifteen years, our research group has been exploring the why Chagas patients do not develop colon tumors within the megacolon segment [10–14]. In two chagasic megacolon patients, we previously reported adenocarcinomas in the nondilated transverse portion of the colon [13]. Analyzing 894 chagasic megacolon cases, we found neither tumors nor preneoplastic lesions, like polyps, within the colonic dilated region [14]. Furthermore, our previous experiments revealed *T*. *cruzi*-infected rats develop less colon tumors than its control group [11]. Chemical myenteric neuronal ablation was also shown protective against the development of colon tumors [15]. Recently, neurons have been suggested to promote prostate cancer, as they enhanced tumor invasion and metastasis [16]. Liebl *et al* have however shown no great impact of neural invasion on colon cancer [17]. These authors then revealed a migration of Schwann cells toward colon tumors occur since early malignant stages [18]. Recently, we discussed how the neuronal activity might promote the development of colon carcinogenesis [19].

Colon carcinogenesis is indeed a complex malignancy and the third most common cancer worldwide [20]. Over 1.2 million patients endure colon cancer in the USA, while prospective data suggest about 150 thousand people will be newly diagnosed *per* year [21]. Colon carcinogenesis develops throughout a multi-stepped sequences of changes [22], from which early lesions transit towards tumors [22–24]. Thus, genetic mutations turn first into detectable preneoplastic lesions before tumors are detected [24]. Bird identified these histological changes in the colon of carcinogen-exposed rodents, naming them aberrant dysplastic crypts (ACF) [23]. Carcinogen-induced changes thus happen in single crypts, which will show altered structure (as width, height, thickness, and luminal opening) due to genomic instability and aberrant cell growth. Researchers have suggested large ACF numbers enhance the risk for colon cancer [25–27].

Current investigation aimed to clarify the why chagasic patients do not develop colon tumors within the megacolon region. We reveal here the myenteric neurons are pivotal elements for the initiation of colon carcinogenesis since its early stages in humans and rats.

## Methods

### Humans

#### Diagnoses and surgery

Surgical ablation together with pathological investigation of twenty-four non-chagasic and chagasic megacolon patients complied the guidelines and approval of ethical institutional review boards (Clinical Hospital of the Federal University of Goias, Brazil; n° 173/2009). After being informed, patients read and signed their consent for collection of colonic samples during surgery.

Chagas megacolon patients were diagnosed based on serological tests against the *T*. *cruzi* (complement protein fixations, passive hemagglutination, indirect immunofluorescence of the *T*. *cruzi*, or ELISA), and X-ray (opaque enema) [13]. Usually, chagasic megacolon was associated with chronic constipation (up to 60 days). Patients underwent colonic surgical resection due to complication in their clinical status (i.e., fecaloma, volvulus, distension, and abdominal pain).

Colon cancer patients underwent surgery for tumor ablation according to the hospitals’ internal guidelines. In all cases, surgical procedures were performed on an elective basis, and consisted of a low anterior resection or Duhamel’s procedure. Codes replace patients’ identification for matters of privacy and confidentiality. Besides a case was either colon cancer or Chagas disease, patient information remained incognito to researchers.

#### Groups

The Brazilian United Health System (SUS; datasus.saude.gov.br) revealed an 80% increase in cancer-related deaths in the whole country from 2005 to 2011 (1,168,011 total cancer-related deaths; 166,858.71 deaths/year). At the same period, colon cancer killed 672 Brazilians at the city of Goias. Considering the latest data from SUS for this Brazilian town (2011–2014), colon cancer death rates were settled at 10.4 and 14.11 deaths/year for males and females, respectively.

Thus, twenty-four random collected colonic samples were divided between two groups of non-chagasic (colon cancer patients; n = 9; age, 70.4±13.9; 78% males and 22% females), and chagasic megacolon cases (n = 15; age, 59.3±10.4; 78% males and 22% females). Samples were collected according to the schedule of the Surgical Division of the Clinical Hospital of the Federal University of Goias for each patient. Inclusion criteria for colon cancer patients were a diagnosis of either rectal or sigmoid non-metastatic adenocarcinomas. Samples were just collected within the surgical safety border (± 5 cm away from the tumor). Only chagasic patients with positive serology for *T*. *cruzi*, diagnosed-megacolon condition, and given above complications in their clinical status were selected. Heading from the operating room to the laboratory, samples were transported in physiological saline solution on ice. Then, samples were rinsed with phosphate-buffered saline (PBS) and fixed in 4% paraformaldehyde solution for 48h. Whereas only samples from the surgical safety border were evaluated in colon cancer patients, dilated-colonic chagasic megacolon region underwent histopathological analysis for each patient just.

### Rats

#### Experiment 1

Male Wistar rats (5 weeks) were housed in a controlled environment within plastic cages (5 rats *per* cage; 55% humidity; 12/12h light/dark; 22+0.5°C). All experiments were performed according to the protocol approved by the Ethical Committee in Animal Care and Use (#59/2014). This approval was based on the guidelines of the National Research Council [28]. Rats were acclimated for one week before starting experiments. They were then divided randomly into the following groups: (1) CTRL (control; n = 8) group received a single saline injection, and was euthanatized in a CO_2_ chamber 24-weeks after. Colon was collected in individual autopsies, and fixed in formalin for 24h. (2) *T*. *cruzi* group (n = 8) was infected with the parasite (10^5^; *T*. *cruzi* Y strain) according to our previous description [29]. Tail blood was weekly collected for the next 12 weeks, from which no more trypomastigotes were found (starting the chronic phase of the disease). Rats were euthanatized at the 24^th^ week from the infection. (3) DMH (1,2-dimethylhydrazine; carcinogen; n = 8) group was weekly exposed to the carcinogen (subcutaneous injection; 20 mg/kg). Rats were euthanatized at the 12^th^ week from the first carcinogenic exposure. (4) *T*. *cruzi*+DMH group (n = 8) was infected with the parasite. Twelve weeks after, rats were weekly exposed to DMH for other 12 weeks. Euthanasia was performed at the 24^th^ week from the infection.

#### Experiment 2

As described for experiment 1, the Ethical Committee in Animal Care and Use (#169/2014) approved other experiments with male Wistar rats. They were randomly divided in the following 6 groups: (1) Sham group (n = 8) underwent anesthesia (1.5% Forane; 98.5% O_2_ [2 L/min]). Then, rats underwent abdominal trichotomy and sterilization (70% alcohol) before a median diaeresis in two planes was performed (cutaneous and muscle planes; 0.5 cm). Colon was exposed out of the abdominal cavity and kept in saline for 30 min. After the colon was replaced into the abdomen, muscle and cutaneous layers were separately sutured closing the abdominal cavity again [15]. Indomethacin (0.2mg x 100g) was given for 48h to avoid pain [30]. Rats were euthanatized at the 6^th^ week from the surgery. (2) BAC (n = 8) group underwent the above described surgical procedure. Instead of saline, the colon was exposed to a chemical denervating buffer for 30 min (benzalkonium chloride, BAC; 2 mM). Before the colon was replaced into the abdominal cavity, it was extensively washed with saline. Indomethacin was then given for 48h. (3) DMH 1 group (n = 8) was exposed to a single carcinogenic DMH injection (125 mg/kg) right after the sham surgical procedure described above (Sham group). Rats were euthanatized after 1 week. (4) DMH 2 group (n = 8) was treated as described for the DMH1 group. Rats were euthanatized after 2 weeks. (5) BAC+DMH 1 group (n = 8) underwent the surgical procedure (BAC group) and carcinogenic exposure (DMH1 group) described above. Rats were euthanatized after 1 week. (6) BAC+DMH 2 group (n = 8) was treated as described the BAC+DMH 1 group. Euthanasia was performed after 2 weeks.

#### Experiment 3

Another experiment was performed with male Wistar rats (#169/2014). They were randomly divided in the following 8 groups: (1) Sham group (n = 8) was treated according to description given above for experiment 2. Rats were then euthanatized 6 weeks later. (2) B group (n = 8) underwent the chemical myenteric denervation as described for experiment 2 (BAC group). After 6 weeks rats were euthanatized. (3) H group (n = 8) endured the Hartmann’s procedure. Before a median diaeresis was performed to expose the colon, rats underwent anesthesia, abdominal trichotomy, and sterilization. Colon was then sectioned up to 10 cm above the rectum. A colostomy was carried out from the proximal portion, while stitches closed the distal colonic segment. Abdominal cavity was closed and anti-inflammatory treatment given as described above for experiment 2. Euthanasia was performed 6 weeks after. (4) HB group (n = 8) underwent the two above described surgical procedures. Rats were euthanatized 6 weeks later. (5) D group (n = 8) was exposed to the carcinogen DMH (125 mg/kg), and euthanatized 6 weeks after. (6) DB group (n = 8) endured the chemical myenteric denervation at the 2^nd^ week following the carcinogenic exposure to DMH. After 4 weeks, rats were euthanatized. (7) DH group (n = 8) underwent the Hartmann’s procedure at the 2^nd^ week following the carcinogenic exposure to DMH. At the 6^th^ week from the DMH exposure, rats were euthanatized. (8) DHB group (n = 8) was exposed to DMH, and, after 2 weeks, rats endured the chemical myenteric denervation and the Hartmann’s procedure. Rats were euthanatized at the 6^th^ week from the DMH exposure.

### Analysis

#### Determination of myenteric neuronal density

Myenteric neuronal density was determined with Giemsa’s special stain, as previously described [31]. Briefly, paraffin embedded samples were sectioned at 7-μm thickness. Sections were then stained with Giemsa’s special stain. Neurons were enumerated within the Auerbach plexus. Either patients or rats had 5 serial tissue sections evaluated. Values are shown as the number of myenteric neurons *per* crypt numbers in each sample.

#### Immunochemistry

Human samples were sectioned at 4 μm thickness, dewaxed, and rehydrated in graded ethanol and distilled water baths. Colon tissue slices were stained with the anti-proliferative cellular nuclear antigen antibody (PCNA; clone PC 10 [1:100; Novocastra, US) according to our standard method for immunochemistry [32]. After overnight incubation at 4°C, Primary antibodies were detected in longitudinal sections with the Picture-MAX Polymer Kit (Invitrogen, USA). Positive reactions were observed a brown precipitate in the nucleus. Proliferating cells were enumerated, values given as positive reactions *per* total crypt cell numbers (iPCNA).

#### Argyrophilic nucleolar organiser regions (AgNOR)

Colonic human sections were stained in silver colloid buffer according to previous description for the agryrophil method [33]. At the crypt bottom, AgNOR dots were enumerated *per* nucleus (immersion oil; 100x magnification). At least 50 crypts were analyzed in each patient.

#### Histopathological analysis for colon preneoplastic lesions

Colon rat samples were stained with hematoxylin and eosin (H&E) according to our previous description [32]. Prior to microscopic analysis, slides were coded to avoid observer bias. At 20x magnification, colon preneoplastic lesions were identified (pathological features ranging from mild to severe dysplasia). Dysplastic features were enumerated at 40x magnification. Colon morphometry (colon sample areas; mm^2^) was performed with the Leica Application Suite (LAS) V3.7 software (Leica Mikrosysteme Vertrieb, Germany). Values for colonic preneoplasia were determined the number of dysplastic lesions *per* analyzed area (mm^2^).

#### Statistical analysis

Data were analyzed using the GraphPad Prism 5 software (Graph Pad Software Inc., US). Mann Whitney test was applied to analyze experiments with two different groups. Experiments with more than two groups were analyzed with the One-way ANOVA test (Dunn’s post hoc test). Two-way ANOVA (Bonferroni’s post hoc test) was applied to analyze different categorical independent endpoints on one dependent variable. Statistical significance was set at *p*<0.05. All values are reported as the mean ± standard deviation.

## Results and Discussion

**Fig 1:**
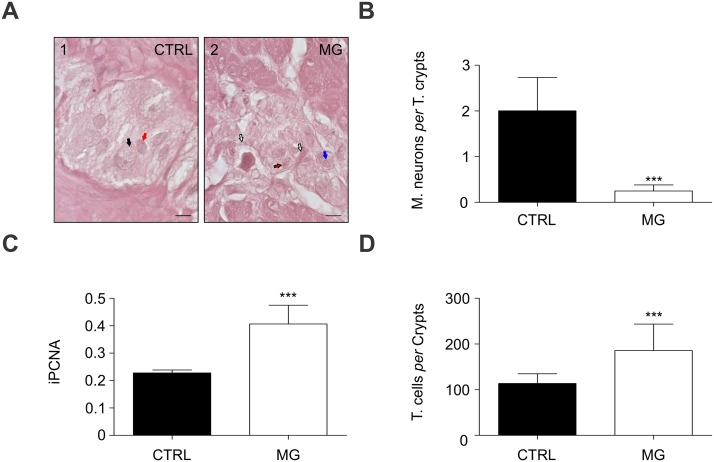
Myenteric neuronal denervation and proliferation in Chagas megacolon. (A) Neurons within the Auersbach’s plexus. Pictures were taken at 100x magnification. Scale bars (10 μm) are inserted. (A.1.) Auersbach’s normal neuronal ganglion is shown within the safety surgical border of an adenocarcinoma patient. Black arrow shows a myenteric neuron, while red arrow leads to a myenteric glial cell. (A.2.) Myenteric neuronal degeneration is shown in a Chagas megacolon patient. Blue arrow shows a myenteric degenerated neuron. Black-white arrows indicate gliosis. Black-red arrow leads to a myenteric degenerated glial cell. (B) Myenteric (M.) neuronal density was determined the number of myenteric neurons *per* total (T.) number of colonic crypts in each sample (***P<0.0001 *vs* CTRL). (C) Proliferation was determined given as positive reactions per total crypt cell numbers (iPCNA; ***P<0.0001 *vs* CTRL). (D) Graph shows the colonocytes numbers *per* colonic crypt (***P<0.0003 *vs* CTRL). Significance was analyzed with the Mann Whitney test. Statistical significance was set at p<0.05. Values are shown as the mean ± standard deviation.

In spite of potential *T*. *cruzi* effects modulating the immune system have been suggested to reduce other tumors besides colon cancer [7–9], how the major *T*. *cruzi* effect on the colon, that is the degeneration of myenteric neurons [11], impacts on colon carcinogenesis are not fully understood yet. Indeed, our latest hypothesis suggests the enteric nervous system play a pivotal role in the colon carcinogenesis [19]. Here, histopathological analysis confirmed a significant myenteric neuronal denervation in chagasic megacolon patients (Fig 1A and 1B).

Such condition was however associated with high-epithelial cell proliferation in the colon (Fig 1C). Also, hyperplastic crypts were largely found in chagasic megacolon patients (Fig 1D).

**Fig 2:**
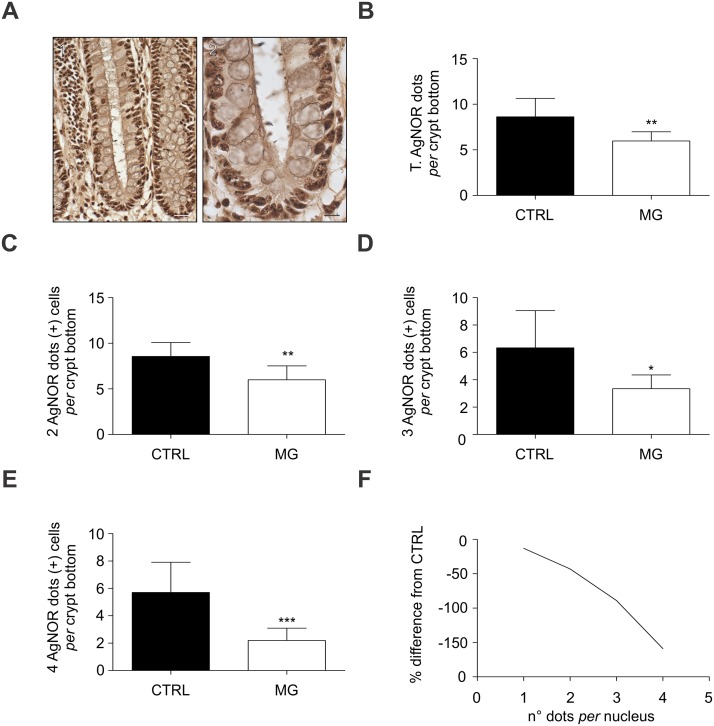
Argyrophilic Nucleolar Organiser Regions (AgNOR) in Chagas megacolon. (A) Illustrative pictures for AgNOR staining in an adenocarcinoma patient. Positive reactions are seen as black dots in the colonocytes nucleus. (A.1) Micrography was taken at 40x magnification (Scale bar = 20 μm). (A.2) Micrography was taken at 100x magnification (Scale bar = 10 μm). (B) Total numbers of AgNOR dots *per* cell nucleus within the crypt bottom are shown (**P<0.0062 *vs* CTRL). (C to E) Total numbers of 2 (C; **P<0.005 *vs* CTRL), 3 (D; *P<0.01 *vs* CTRL), and 4 (E; ***P<0.0001 *vs* CTRL) AgNOR dots (+) cells *per* crypt bottom are shown. Significance was analyzed with the Mann Whitney test. Statistical significance was set at *P*<0.05. Values are shown as the mean ± standard deviation. (F) Mean values for percentage difference between megacolon and adenocarcinoma samples are shown according to nuclear distribution of AgNOR dots *per* nucleus.

Analyzing the AgNOR staining within the cryptal bottom revealed however reduced risk for colon cancer in Chagas megacolon patients (Fig 2A and 2B).

Next, another analysis was performed to enumerate the number of AgNOR dots per cell nucleus. Here, we found as much dots a cell had, as much rare it was found in chagasic megacolon (Fig 2C and 2D).

**Fig 3:**
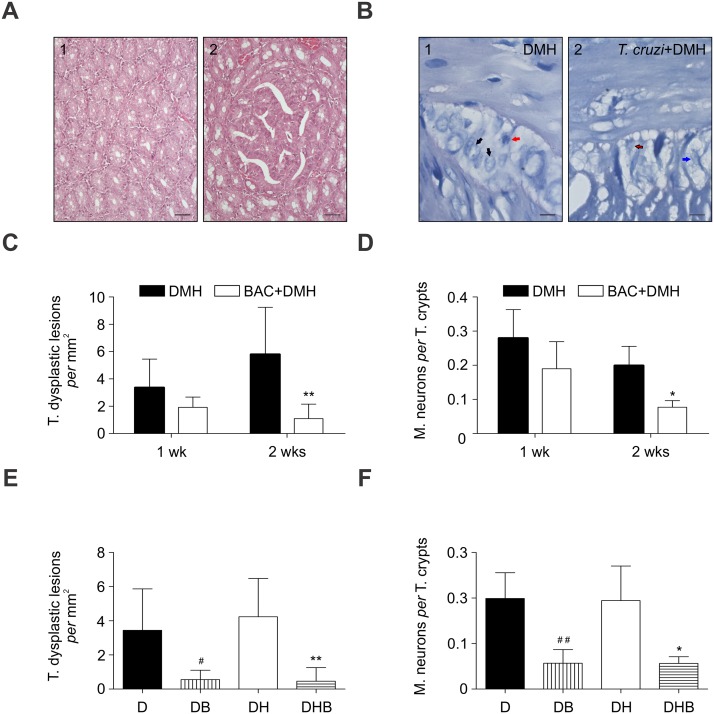
Myenteric neuronal denervation and colon preneoplastic lesions in rats. (A) Illustrative pictures for normal (A.1) and preneoplastic (A.2; severe dysplasia) colon tissues from a carcinogen-exposed rat. (B) Illustrative pictures for normal (B.1) and degenerated (B.2) myenteric plexus in uninfected (B.1) and infected *T*. *cruzi* rats (B.2). Black arrow shows a myenteric neuron, while red arrow leads to a myenteric glial cell. Blue arrow shows a myenteric degenerated neuron. Black-red arrow leads to a myenteric degenerated glial cell. (C) Total (T.) numbers of colon dysplastic lesions *per* mm^2^ are shown in carcinogen-exposed groups (DMH, 1,2 dimethylhidrazine; BAC, benzalkonium chloride; **P<0.01 *vs* DMH). (D) Myenteric (M.) neuronal density was determined the number of myenteric neurons *per* total (T.) number of colonic crypts in each sample (*P<0.05 *vs* DMH). Significance in the C and D graphs was analyzed by the Two-way ANOVA with Bonferroni’s posttest. (E) T. numbers of colon dysplastic lesions *per* mm^2^ are shown in carcinogen-exposed groups (^#^ *P*<0.05 *vs* D; **P<0.01 *vs* DH). (F) M. neuronal density was determined the number of myenteric neurons *per* T. number of colonic crypts in each sample (^##^ *P*<0.01 *vs* D; *P<0.05 *vs* DH). Significance in the E and F graphs was analyzed by the One-way ANOVA with Dunn’s posttest. Statistical significance was set at *P*<0.05. Values are shown as the mean ± standard deviation.

Ascertaining the relationship between myenteric neuronal activity and colon carcinogenesis, *in vivo* experiments were performed. Rats were weekly exposed to a carcinogen after 86-days from *T*. *cruzi* infection. Following those 12-wks of carcinogenic exposure, *T*. *cruzi*-infected rats developed significant less colon preneoplastic lesions than their uninfected control group (Fig 3A; DMH, 2.89 ± 2.1 vs *T*. *cruzi*+DMH, 1.05 ± 0.94 T. dysplastic lesions per mm^2^; *p*<0.04).

Interestingly, a significant colonic myenteric neuronal denervation was observed in that carcinogen-exposed and *T*. *cruzi*-infected group (Fig 3B; DMH, 0.15 ± 0.04 vs *T*. *cruzi*+DMH, 0.05 ± 0.01 M. myenteric neurons *per* T. crypts; *p*<0.0001). No preneoplastic lesions were observed in rats that did not undergo carcinogenic exposure.

Then, we set out to investigate whether this modulation on myenteric neuronal density plays a pivotal role during the early steps of colon carcinogenesis. A well-established chemical denervation model was applied in carcinogen-exposed rats [34]. Puzzling, preneoplastic lesions were found decreased from the 2^nd^ wk onward (Fig 3C), which ensued having the colon myenteric denervation significantly induced (Fig 3D).

Yet, Hartmann's procedure was performed to clarify the activity of myenteric neurons aside from the colonic fecal content. With low mortality rates in humans [35], such procedure results in an end colostomy and colonic stump closure. Having the carcinogen exposure before surgery, preneoplastic lesions develop without any potential effects from the fecal content on them. Again, colon preneoplastic lesions developed significantly less in myenteric denervated rats (Fig 3E and 3F).

Our collective data demonstrate myenteric neurons are a key-factor for the development of early carcinogenic lesions in the colon. Neuronal activity has been previously shown essential for the colonic homeostasis. For instance, *NSE-noggin* and *Hand2* ^*+/-*^ mice (transgenic models with more or less enteric neural innervations than normal, respectively) were exposed to dextran sulfate sodium to induce intestinal inflammation. *Hand2* ^*+/-*^ mice showed much lower inflammatory signals than wild-type littermates, which did not occur in *NSE-noggin* mice [36]. However, colitis was previously observed in chagasic megacolon patients [37], as well as such condition is a well known risk factor for colon cancer [38]. Interestingly, we found here no tumors within the megacolon segment.

*T*. *cruzi*-infected patients undergo deep and complex modulations throughout the development of megacolon. For instance, an acute transitory immunosuppression has been found in *T*. *cruzi*-infected patients [39,40], in spite of colonic enteric ganglia undergo unremitting cytotoxic T cell invasion for the chronic development of chagasic megacolon [41]. Considering that regardless of infectious agents chronic inflammation promotes colon cancer [42], the most stable factor known to promote colon cancer but missing in chagasic megacolon is the myenteric neurons. This indeed supports our previous hypothesis that colonic neurons exposed to a carcinogen might enhance the development of carcinogenic initiated colonocytes into preneoplastic lesions [19].

Despite a neural invasion has not been observed in colon tumors [17], as reported in prostate cancer [16], the role of neuronal elements in the colon carcinogenesis cannot be ruled out [18]. Puzzling, reports have shown neuronal denervation leading to megaesophagus did not protect chagasic patients from the development of malignancies at this gastrointestinal upper region [43,44]. Despite such data appear controversial at first it actually supports the idea of a neuronal activity specifically promoting preneoplastic lesions in the colon. Actually, a specific stimulus could induce distinct effects in different body tissues. For instance, human antigen R (HuR) has different activities in azoxymethane-exposed small bowels and colon tissues; whereas it enhances prosurvival gene expression in the small bowels, colonic epithelial HuR downregulates specific proapototic RNAs in carcinogen-exposed mice [45]. Thus, we also showed myenteric neurons promote the development of colon tumors in two different experimental models [11,15]. Considering our latest hypothesis that epithelial cells and neuronal elements might undergo carcinogenic changes in the colon tissue [19], current investigation demonstrate that the neuronal activity is essential for the development of early colonic carcinogenic events.

Taken together, chagasic megacolon seems a natural model to study how the enteric nervous system impacts on colon tissue. We thus believe myenteric neurons promote the colon carcinogenesis since its early stages. This might clarify the why tumors do not develop within the chagasic megacolon region. It seems reasonable to suggest that besides the direct effects of a carcinogen on colonic epithelial cells, neuronal activity is also required for the full development of colon preneoplastic lesions.
